# Magnetic resonance imaging-guided occult breast lesion localization and simultaneous sentinel lymph node mapping

**DOI:** 10.1186/1477-7819-12-320

**Published:** 2014-10-23

**Authors:** Marcos Fernando de Lima Docema, Paulo Aguirre Costa, Felipe Eduardo Martins de Andrade, Jose Luiz Barbosa Bevilacqua, Simone Elias, Giovanni Guido Cerri, Alfredo Carlos SD Barros, Afonso Celso Pinto Nazario

**Affiliations:** Magnetic Resonance Imaging Department, Hospital Sírio Libanês, Rua Dona Adma Jafet, 91, Bela Vista, São Paulo, SP 01308-000 Brazil; Nuclear Medicine, Hospital Sírio Libanês, Rua Dona Adma Jafet, 91, Bela Vista, São Paulo, SP 01308-000 Brazil; Mastology Studies Department, Hospital Sírio Libanês, Rua Dona Adma Jafet, 91, Bela Vista, São Paulo, SP 01308-000 Brazil; Discipline of Mastology, Escola Paulista de Medicina, Universidade Federal de São Paulo (UNIFESP), Rua Botucatu 740, Vila Clementino, CEP 04023-062 São Paulo, SP Brazil; Hospital Sírio Libanês, Rua Dona Adma Jafet, 91, Bela Vista, São Paulo, SP 01308-000 Brazil; Imaging Diagnostics Department, Hospital Sírio Libanês, Rua Dona Adma Jafet, 91, Bela Vista, São Paulo, SP 01308-000 Brazil

## Abstract

**Background:**

Radio-guided occult lesion localization is a valid technique for the diagnosis of suspicious non-palpable lesions. Here we determine the feasibility of pre-operative localization of occult suspect non-palpable breast lesions using radio-guided occult lesion localization, as well as for identifying the sentinel lymph node.

**Methods:**

This is a descriptive study of data collected retrospectively. Pre-operative mapping of 34 breast lesions in 25 patients suspected of being malignant was performed using conventional imaging methods with a magnetic resonance imaging-guided radiopharmaceutical injection.

**Results:**

The mean time required to perform the localization was 25 minutes. After resection of the lesions using a gamma probe, malignancy was confirmed in fifteen patients (60.0%), with nine invasive ductal carcinomas, two invasive lobular carcinomas, and four *in situ* ductal carcinomas The resection was confirmed by the complete removal of the radioactive material. The pathologic results and images were concordant in all but two cases, which were submitted for new magnetic resonance imaging examinations and surgery that confirmed the malignancies. Of the 15 patients with confirmed malignancies, 10 had sentinel lymph node resection. Of these, eight were negative for metastases, one had micro-metastases and one had confirmed metastases. Three patients had full axillary node dissection, with metastases found in only one. No side effects were observed with magnetic resonance-guided radiopharmaceutical injection.

**Conclusions:**

The sentinel node occult lesion localization technique is a simple, reproducible and effective alternative approach to occult lesions compared to other methods, such as mammotomy and the hook-wire localization technique, for mapping suspect breast lesions and identifying lymph node metastasis.

## Background

Radio-guided occult lesion localization (ROLL), described in 1998, is a suitable technique for the removal of suspicious non-palpable breast lesions (NPLs) [1, 2]. The procedure requires injection of dextran conjugated to technetium (^99m^Tc) directly into the area to be resected, guided by ultrasound or mammographic stereotactic localization [3]. Although the hook wire-guided method remains the most employed technique for surgical biopsies of NPLs, ROLL is used progressively more often worldwide for open surgery biopsy. The injection of a radiopharmaceutical allows precise pre-operation localization of subclinical abnormalities and eliminates some of the inconveniences of a wire localization [4]. ^99m^Tc-dextran applied locally to the primary tumor is taken up through the lymphatic system and accumulates in the lymph node through phagocytosis. Nevertheless, most of the injected dosage remains in the injection site. Radioactivity retention allows localization of the tumor with the help of a radiation probe [5].

It is well known that sentinel lymph node biopsy can accurately predict the presence or absence of axillary lymph node metastasis in patients with early-stage infiltrating breast carcinoma, and these types of lesions are suitable for both radioisotopic localization and radioguided sentinel lymph node biopsy, as previously described elsewhere (sentinel node occult lesion localization; SNOLL) [2].

Approximately 10% of malignant lesions in the breasts are detected exclusively by contrast material-enhanced magnetic resonance imaging (MRI) [6]. Subsequent comparative evaluation of the methods, with ultrasound examinations or a new mammography reading based on MRI information, demonstrated that approximately half of the cases are resolved, leaving the other half with exclusively MRI-based findings. How to approach these lesions is quite a dilemma, since biopsy devices or MRI-based lesion-localization devices are not easily available because of high costs. The objective of this study is to describe a new preoperative mapping technique for NPLs suspected of being malignant, which are occult, using conventional imaging methods, with a magnetic resonance-guided radiopharmaceutical injection, which is simultaneously capable of identifying the sentinel lymph node, all in a single procedure guided by MRI.

## Methods

### Subjects

This is a descriptive study of data collected retrospectively. This study was approved by our institutional medical ethical review board (CEPesqHSL2008/46). All patients provided informed consent, using an approved consent form. Patients were selected from a group that had undergone breast MRI between October 2007 and June 2008 and had suspicious incidental MRI findings which were occult by conventional methods such as physical examination, mammography and ultrasound. The average age of the patients was 53.1 years (range 33 to 70 years). The patients included in this study did not have any contraindications to inclusion in the MRI study (for example, permanent pacemaker implanted, cerebral aneurysm clip made of non-compatible material or bilateral hip prosthesis) or to the use of paramagnetic contrast agents (for example, renal insufficiency or allergic reaction to gadolinium). All patients selected had their mammography reviewed, and underwent a new, guided ultrasound examination, thereby confirming the finding exclusively by MR. None of the patients had clinical suspicion of axillary lymph node tumors.

The lesions were classified according to the lexicon established by the American College of Radiology [7].

### Magnetic resonance imaging

We analyzed retrospectively the pre-surgical MRI-guided radiopharmaceutical injection and breast mapping of 34 lesions of 25 patients, suspected to be malignant and occult under conventional imaging methods. The procedures were implemented using 3 T equipment (Signa HDX; General Electric Medical Systems, Milwaukee, WI, USA), an 8-channel dedicated coil, with lateral and medial openings.

The procedure was performed the day before, or on the morning of, the surgery (allowing at least a 3-hour interval). All patients had MRI performed at our department, with clinically, mammography and sonography occult findings of suspected malignancy and uncompromised axillary nodes. With the patient in the ventral decubitus position with compression of only the involved breast, we conducted the dynamic study by applying an endovenous injection of a paramagnetic contrast agent (0.1 mmol per kg; OptiMark, Tyco Healthcare, Mallinkrodt – St. Louis, MO 63042 U.S.A.) and then performing a T1-weighted FLASH (Fast Low Angle Shot) three-dimensional pulse sequence to take images 1 mm thick. One sequence before, and two sequence after, intravenous contrast injection, with subsequent imaging subtraction from pre-contrast images on a pixel-by-pixel basis by diagnostic workstation. After identifying the lesion in the computer, the spatial coordinates were noted (X, Y and Z axes) and its relation to the nipple spatial coordinates.

Using a lateral cross grid (2 × 2 cm) we measured the distance from the lesion to the nipple. Based on the lesion coordinates, it was possible to determine the projection of the lesion on the skin within the cross grid and mark it with a vitamin E capsule, which appears in the dynamic study with contrast agent. One of the dynamic study sequence was repeated until the marker was exactly in the direction of the lesion (X and Y coordinates adjusted), with only the distance to the skin remaining to be measured, which corresponded to the length of the needle to be introduced (Z axis).

The antisepsis was made with alcoholic iodine, then anesthesia was performed with 1 ml 2% liquid lidocaine without a vasoconstrictor.

**Figure 1:**
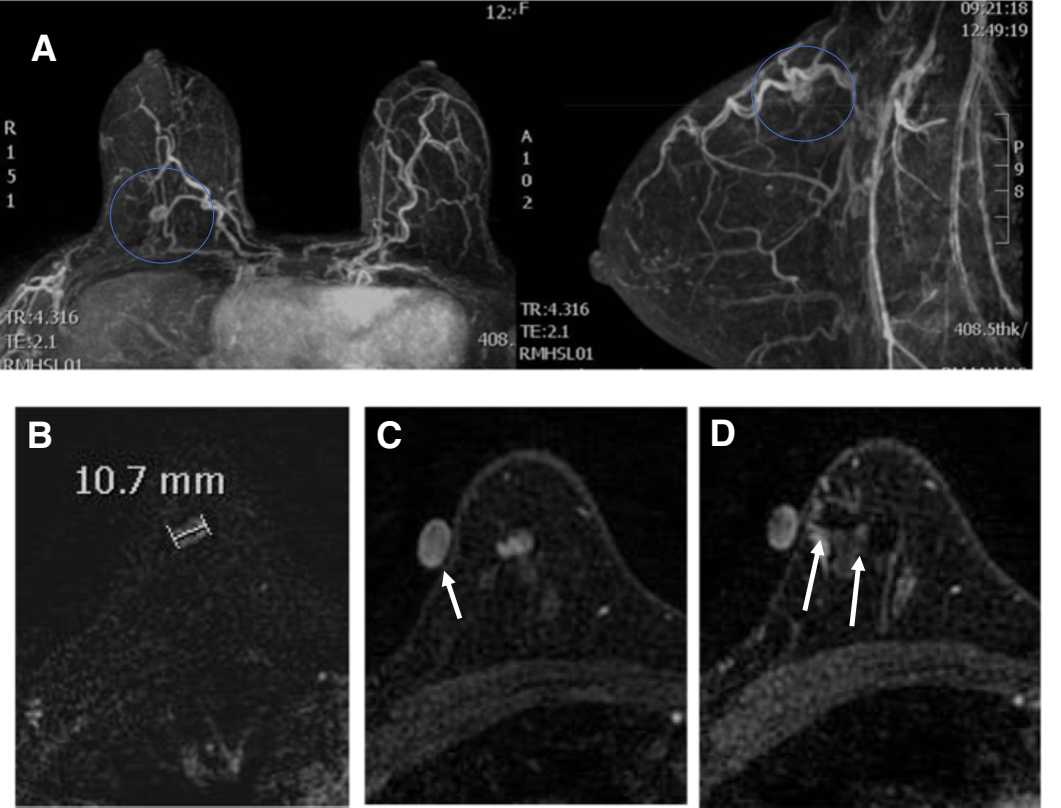
**A 44-year-old woman with normal mammographic and sonographic findings.** Histological diagnosis of infiltrating lobular carcinoma. **(A)** Transverse and sagittal maximum intensity projection (M.I.P) reconstruction of contrast-enhanced dynamic study of a suspicious lesion in the upper quadrants of the right breast (circle). **(B)** Transverse T1-weighted contrast-enhanced subtracted magnetic resonance image showing the lesion. **(C)** The relationship between the skin surface and the vitamin E marker (arrow). **(D)** Compatible needle and free-contrast artifacts covering the lesion (arrows).

A needle made of MR-compatible material (titanium alloy, 20-gauge EZE-M) is usually employed, but the use of such a needle has only recently been allowed by the regulatory authorities in Brazil. We developed an alternative method using an intravenous catheter (BD Insyte Autoguard, Becton Dickinson Ind. Cir. Ltda, Juiz de Fora, MG, 36081-000, Brazil), which can be visualized when mapping the lesions without producing magnetic susceptibility artifacts, and then removing the metallic guide and filling the catheter with a paramagnetic contrast agent diluted in a 0.9% saline solution. After the needle was introduced the pulse sequence was repeated for position control until the needle had reached the lesion’s periphery Once the correct positioning of the needle at the periphery of the lesion was confirmed, we injected, into the target area, a trace amount (0.1 ml) of radioactive material for conventional medical purposes (in this case, 0.5 mCi pharmaceutical grade dextran 500 conjugated to ^99m^Tc) (Figure 1).

**Figure 2:**
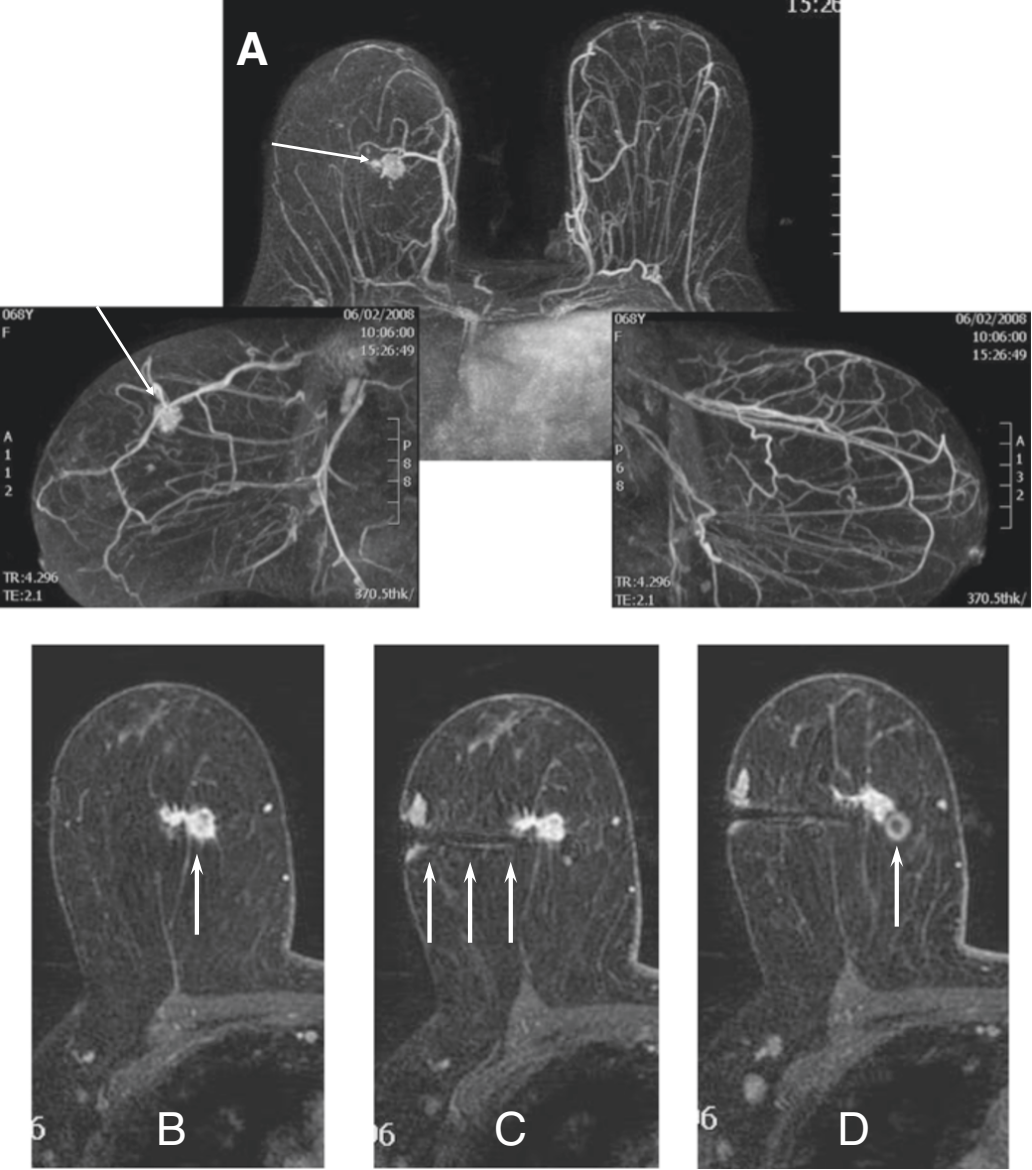
**Steps of radiopharmaceutical injection at a suspicious MRI finding. (A)** 3D MIP reconstruction contrast-enhanced dynamic study of a bilateral breast magnetic resonance with parallel imaging technology, performed on a suspicious mass in the right breast (arrows). **(B)** Transverse T1-weighted dynamic contrast-enhanced image showing the upper inner quadrant irregular mass (arrow). **(C)** Compatible needle near the target lesion (arrows). **(D)** Perilesional injection reveals an artifact from a small quantity of gadolinium to confirm the location of the injection (arrow). A 52-year-old woman whose excisional biopsy findings revealed a diagnosis of infiltrating ductal carcinoma.

The accuracy was determined on the basis of the needle extremity being 1 cm or less from the target lesion (Figure 2). A small amount of gadolinium diluted in solution (0.1 ml gadolinium per 0.4 ml 5% saline solution), together with the ^99m^Tc-dextran, was used to confirm the location of the injection in a pulse sequence performed after the needle was removed.

**Figure 3:**
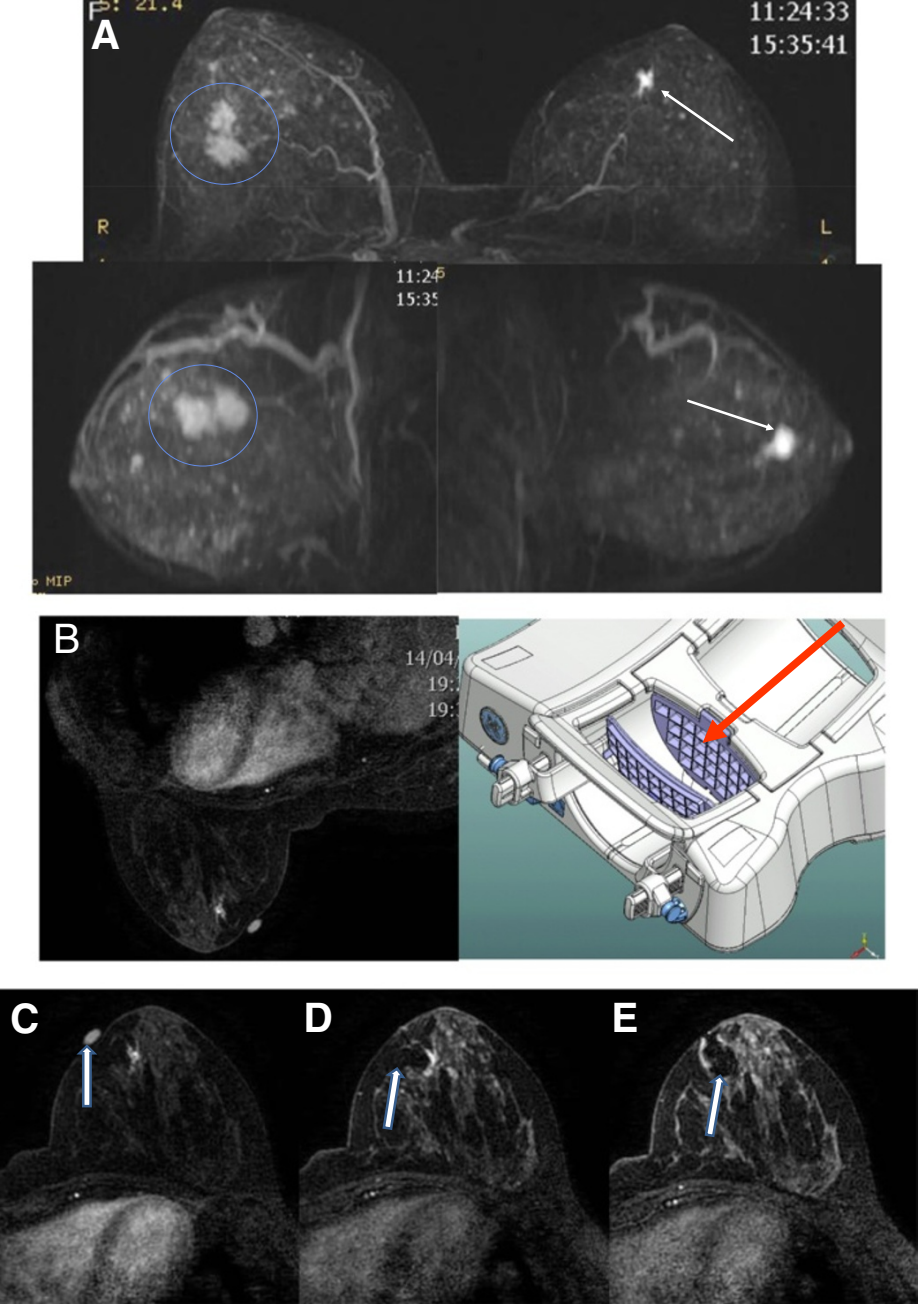
**How medial access of a distant lesion may be performed. (A)** A 58-year-old woman recently diagnosed with cancer of the right breast (circles). Magnetic resonance imaging revealed a 6-mm sonographically and mammographically occult contralateral breast lesion (arrow). **(B)** Medial accessibility: medial breast lesion may be approached from the opening in the contralateral space coil (arrow). **(C)** Medial vitamin E capsule near the lesion (arrow). **(D)** Compatible needle (arrow). **(E)** Contrast artifact covering the lesion (arrow). Histological analysis revealed an atypical ductal hyperplasia with infiltrating ductal carcinoma in the contralateral breast.

**Figure 4:**
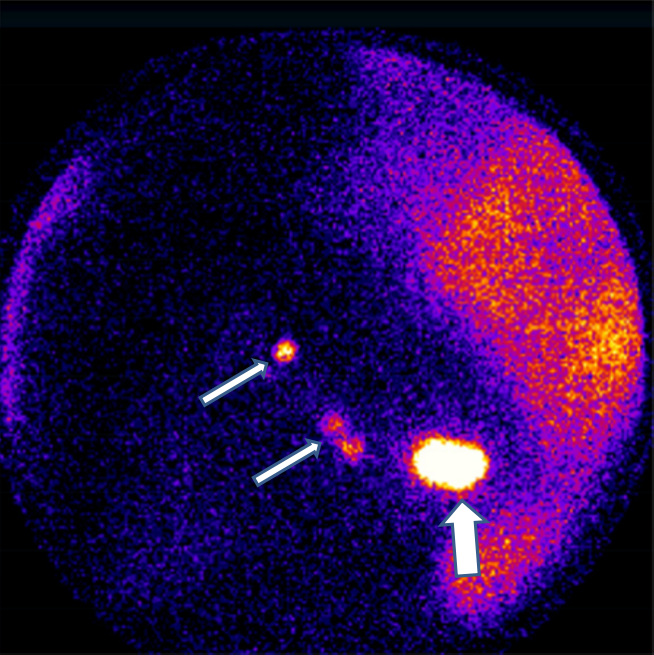
**Scintigraphic image (lateral projection) showing the area of the injection in the breast (thick arrow) and the axillary sentinel lymph nodes (thin arrows).**

Lesions located in the external quadrants were approached from the corresponding lateral access. Medial lesions were approached from the access in the contralateral coil, raising the opposite breast, and using the coil space to reach the medial face of the breast in question (Figure 3). Scintigraphic control images were taken an average of 3 hours after the injection of the ^99m^Tc-dextran using a scintillation camera (Siemens/E-CAM, gamma camera, Siemens AG Healthcare Sector, Henkestrasse 127, 91052 Erlangen, Germany); collimation 140 keV and 300,000 counts in anterior and lateral projections), identifying the area in question in the breast and the sentinel lymph node (Figure 4). The surgical dissection of the lesion following the injection (after a minimum interval of 3 hours) was guided plane-by-plane using a radiation probe (Crystal gamma-probe, Nuclear Fields USA Corp, 1645 S. River Road Suit 5, Des Plaines, Il- 60018 U.S.A.) that accurately determined the volume to be resected, decreasing morbidity and surgical time. A margin of 1.0 cm around the suspect lesion or the removal of all radioactive material was respected [8]. When a malignancy was found, adequate resection was performed with intraoperative evaluation of the margins pursuant to standard protocol [8]. In cases of invasive or ductal carcinoma *in situ* (DCIS) with a high degree of comedonecrosis, the procedure was complemented with sentinel lymph node biopsy guided by the gamma-probe.

## Results

The lesions were classified according to the lexicon established by the American College of Radiology [7]. One lesion was classified as breast imaging reporting and data system (BI-RADS) 6, as it was a residual node found after neo-adjuvant chemotherapy; nine were classified as BI-RADS 5; and seventeen as BI-RADS 4. Seven lesions that were classified as BI-RADS 3 were marked because of their association with a B5 lesion on the opposite breast. The size of the lesions ranged from 0.5 to 1.8 cm (mean 0.99 cm).

With respect to localization, 18 lesions were found in the right breast and 16 in the left breast, with bilateral lesions in two patients. Of the 34 lesions, 29 (85.3%) were mapped by lateral access, making the procedure easier; in the five other lesions, access was medial. There was satisfactory mapping of the site of the lesion and of at least one sentinel lymph node in all lesions. After gamma-probe guided resection of the lesions, malignant tumors were confirmed in 15 patients (60.0%); nine with invasive ductal carcinoma, two with invasive lobular carcinomas and four with DCIS. Of these, ten underwent resection of only the sentinel node, which was analyzed during the operation, as the broad axillary approach was unnecessary. The other two patients, who had low grade DCIS, did not have sentinel lymph node resection. In the other three patients, submitted to formal axillary dissection, the sentinel lymph node was mapped, and was positive for metastases in one case, but negative in the other two. Other histological findings included a complex sclerosing lesion, radial scar, fibroadenoma, hamartoma and ductal hyperplasia as cited at Table 1.

**Table 1:** **Characteristics of breast lesions, lesion size, histologic findings, and lymph node status**

Patient number	Age (years)	BI-RADS	Size (mm)	Is the main histological finding?	Additional MRI finding	Histologic finding	Lymph node status: Metastasis/resected
1	44	5	13	Yes		ILC	1/22
2	46	4	10	Yes		FA	
3	53	4	10	Yes		IDC	0/3
4	42	4	5	Yes		CSL	
5	59	5	8	Yes		IDC	0/1
6	49	6	13	Yes	PNC	IDC	0/34
7	50	3	8	No	IDC Contralateral	DH	
8	52	3	7	Yes		DH	
9	48	3	10	Yes		CSL	
10	70	5	18	Yes		IDC	1/2 MI
10	70	3	7	No	B5 Contralateral	DH	
11	55	4	5	Yes		FN	
12	68	5	17	Yes		ILC	0/11
12	68	3	8	No	B5 Contralateral	SF	
13	52	5	10	Yes		Breast Tissue	
13	52	5	10	Yes		IDC	0/1
14	54	4	15	Yes		CSL	
14	54	4	11	Yes		CSL	
15	33	4	12	Yes		DH	
16	60	4	15	Yes		Papilloma	
16	60	4	10	Yes		Papilloma	
17	31	4	9	Yes		DCIS	0/1
18	60	5	11	Yes		IDC	1/3.
18	60	4	7	Yes		IDC	0/2
19	62	4	8	Yes		DCIS	Low-Grade
20	66	5	12	Yes		Breast Tissue	
20	66	5	12	Yes		IDC	0/1
21	29	4	12	Yes		H	
22	45	4	8	Yes		DCIS	Low-Grade
23	46	4	5	No	B5 Ipsilateral	IDC Multicentric	0/1
23	46	4	7	No	B5 Contralateral	CSL	
24	37	3	5	No	B5 Contralateral	SF + IDC Contralateral	
25	68	4	7	No	IDC Ipislateral	DCIS + IDC	0/3
25	68	3	5	No	IDC Contralateral	DH	

BI-RADS, breast imaging reporting and data system; CSL, complex sclerosing lesions; DCIS, ductal carcinoma *in situ*; DH, hyperplasia ductal without atypia; FA, fibroadenoma; FN, fat necrosis; H, hemangioma; IDC, infiltrating ductal carcinoma; ILC, infiltrating lobular carcinoma; stromal fibrosis.

The mean time to map the lesions using MR equipment was 25 minutes. The distance between the skin and the lesions ranged from 1.0 to 4.5 cm (mean 2.8 cm). In only one lesion we had to relocate a second needle in a position that was satisfactory along the periphery of the defined target. No intercurrences were observed. Confirmation of resection was established by the complete removal of the radioactive material. The pathological results and the images were concordant in all cases, except in two patients. In those two patients, a new MRI examination was performed 28 days after surgery, and persistent lesions were found near the resection. Both underwent surgery, and malignant lesions were found. Two patients who had multicentricity underwent full mastectomy. All other patients had a new MRI examination 6 months after surgery and did not have any detectable lesions.

## Discussion

MRI, incorporated in the pre-operative approach to breast cancer, is used to control the response to neo-adjuvant chemotherapy treatment, to investigate occult breast carcinomas, and to differentiate recurrence from glandular or scar tissue alterations. In addition, it has been validated in multicentric studies for screening high-risk patients [9, 10]. As this is a highly sensitive method, based on anatomical and functional information, it often reveals alterations suspected of malignancy that do not appear in clinical tests or are not well defined by conventional imaging methods. Thus, MRI is the best tool for locating and approaching these lesions, which generally represent tumors in their initial stages for which segmented resection and biopsy of the sentinel lymph node are favorable, given the low risk of axillary metastasis.

Wiener and colleagues [11] studied the value of using breast MRI with a contrasting agent in planning conservative surgery, compared to mammography and ultrasound. MRI detected one or more cancer foci in the same breast in 32% of patients, and in the contralateral breast of 9% of patients [11]. The presence of various cancer foci in the same quadrant is associated with a greater risk of local recurrence and can require a more ample excision or the disregarding of conservative surgery [12, 13]. Liberman and colleagues [14] identified a second cancer focus by MRI of the ipsilateral breast in 27% of patients that had not appeared before in the mammography and physical examination. Multicentricity and multifocality were more common in patients with a family history of breast cancer (42% vs 14%) and in women where the primary tumor was an invasive lobular carcinoma (55% vs 22%) [14].

Fischer and colleagues [6] demonstrated that MRI exclusively detected 6.5% of multifocality, 5.3% of multicentricity, and 3.2% of additional contralateral carcinoma. These results produced a correct change in surgical and therapeutic approach in 14.3% of the cases [6]. Berg and colleagues demonstrated that MRI revealed additional foci, which resulted in a change in surgical strategy in 30% of the patients. Bilaterallity was seen in 9% of these patients [15].

Finding a lesion using MRI that was not found using ultrasound or mammography is common in our daily practice. Teifke and colleagues [16] concluded that the incidental lesions detected using MRI should be re-evaluated using ultrasound or mammography. If those lesions were not identified using these two techniques, they should be subject to biopsy by MRI, when suspect, or be reviewed every 6 months, when probably benign [16].

Brown and colleagues observed incidental imaging in 29% (30/103) of patients with a negative mammography [17]. Nearly 10% of malignant lesions appeared exclusively under MRI, according to Fischer and colleagues [6].

Although mammotomy is still the best option to exclusively access MRI-identified suspicious breast lesions, it is not available in all medical centers throughout world and has high costs. In those centers where mammotomy is not available, other alternatives are required to access these kinds of lesions.

Our article proposes a new technique to approach these suspicious lesions, with a radio-pharmaceutical injection and its posterior surgical removal, thus suggesting a viable and effective alternative with minor discomfort compared to conventional hook wire-guided techniques used in centers where mammotomy is not available.

The radio-pharmaceutical injection can be used in other situations, such as in patients with mammary prosthesis who have suspicious lesions on the MRI located in close contact to the implant, which can be damaged by a fragment biopsy.

Conservative surgery can be performed in patients who have advanced breast cancer and had been submitted for neo-adjuvant chemotherapy having reached a good response with tumor reduction that resulted in NPLs that were identified exclusively by MRI. In these cases, our technique can be performed to identify the residual tumor for posterior conservative resection with safety margins.

Women who had been diagnosed with a malignant breast cancer and are candidates for conservative surgery, and who have been identified with a new suspicious lesion in the same or contra-lateral breast at the pre-surgical MRI, are another group of patients who can benefit from this technique. A significant number of these lesions are only identified by MRI; a new MRI-guided mammotomy can be performed in these cases, but this has an impact on costs and time, retarding surgery. Considering that surgery will always be performed, the radio-pharmaceutical marking of these lesions can be performed, allowing the surgeon to address this finding during procedure by performing a frozen biopsy. If malignancy is confirmed, the surgeon can immediately review his strategy, and the radio-pharmaceutical can still be used to locate the sentinel lymph node if it is an invasive cancer.

Our data corroborates the paper of Yilmaz [18], who described a technique that successfully marked suspicious NPLs with technetium. However, unlike those authors who used a commercially available dedicated biopsy compression device, a disposable cannulation needle block and a specific software that gave the coordinates of the lesions, we developed a simple, low-cost and reproducible technique.

Pereira and colleagues [19] also described a technique that successfully marked suspicious NPLs with technetium, but we did not need to use a titanium needle to inject the radio-pharmaceuticals. We developed this new low-cost alternative at a time when there was no access in Brazil to titanium needles. The goal of our work was to develop an alternative for medical centers that do not perform MRI-guided mammotomy. In these cases, the costs of the procedure would be also important, therefore the use of an intravenous catheter (BD Insyte Autoguard) reduces the costs and the need for a titanium needle. The other difference from the technique of Pereira and colleagues is that we did not have the need to anchor the main needle with another one, reducing patient discomfort and avoiding complications. We created a ‘MRI-compatible needle’ with common and widely available material found in hospitals and clinics.

We also demarcated the injection site with a small amount of gadolinium, prior to the radio-pharmaceutical injection. This demarcation with gadolinium can be performed more than once until the right spot of the lesion is reached. This paramagnetic contrast is easily identified in the subsequent acquired images, without further need for imaging subtraction.

Moreover, we did not inject distilled water as was proposed by Pereira and colleagues, reducing the dilution and dispersion of the radio-pharmaceutical. In our data, we did not have any case of radio-pharmaceutical dispersion or lack of capture by the sentinel lymph node, confirmed by scintigraphy.

The radio-pharmaceutical used in our paper was Dextran 500 conjugated to ^99m^Tc due to its stability, reproducibility and biological safety. Moreover, we have been using this radio-pharmaceutical at our department for a long time. We also opted for scintigraphy performed after 3 hours of injection, avoiding any failure in the capture of the sentinel lymph node which can happen due to the migration delay of the radio-pharmaceutical in mainly elderly or obese patients.

The dextran 500 conjugated to ^99m^Tc allows the marking and radio-guided resection of non-palpable lesions (ROLL) and, at the same surgical time, resection of the sentinel lymph nodes (SNOLL) in those patients with confirmed malignancy.

## Conclusion

Combining ROLL and sentinel lymph node biopsy in a single procedure (SNOLL), using radiopharmaceutical injection guided by ultrasound or mammographic stereotactic localization, is a precise and well- established method; however, these methods do not access the occult breast cancer. We describe a new, safe and feasible MRI technique that has successfully marked suspicious NPLs and enabled their radioguided surgical resection. In addition, our technique allows the resection of the sentinel lymph node at the same surgical time. To our knowledge, it is the first time a technique like this has been described.

Larger studies are needed to determine the sensitivity, specificity and positive and negative predictive values of this new diagnostic resource.

## Abbreviations

BI-RADS: breast imaging reporting and data system
MRI: magnetic resonance imaging
NPL: non-palpable breast lesion
ROLL: radio-guided occult lesion localization
SNOLL: sentinel node occult lesion localization.
